# A strategy for orthogonal deubiquitination using a bump-and-hole approach†

**DOI:** 10.1039/d3cb00095h

**Published:** 2023-10-02

**Authors:** Takumi Suzuki, Yuki Utsugi, Satoshi Yamanaka, Hirotaka Takahashi, Yusuke Sato, Tatsuya Sawasaki, Yusaku Miyamae

**Affiliations:** a Master's/Doctoral Program in Life Science Innovation, Graduate School of Comprehensive Human Sciences, University of Tsukuba Tsukuba Ibaraki 305-8572 Japan; b Division of Cell-Free Science, Proteo-Science Center, Ehime University Matsuyama Ehime 790-8577 Japan; c Center for Research on Green Sustainable Chemistry, Tottori University Tottori 680-8552 Japan; d Department of Chemistry and Biotechnology, Graduate School of Engineering, Tottori University Tottori 680-8552 Japan; e Institute of Life and Environmental Sciences, University of Tsukuba Tsukuba Ibaraki 305-8572 Japan miyamae.yusaku.fw@u.tsukuba.ac.jp

## Abstract

We have successfully applied a bump-and-hole approach to establish orthogonal deubiquitination in which a ubiquitin substrate variant is specifically targeted by an engineered deubiquitinating enzyme (DUB). This makes it possibe to selectively observe and measure a single type of DUB activity in living cells.

## Abstract 2

We established orthogonal deubiquitination, which enables us to selectively observe a single type of deubiquitinating enzyme activity in living cells.

## Graphical abstract

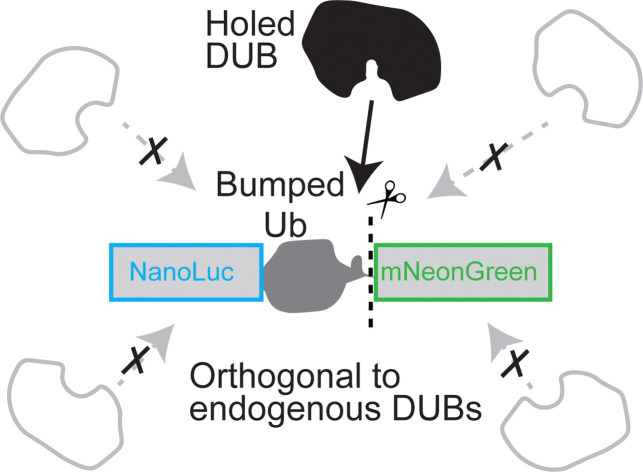

Cellular protein abundance is tightly regulated by degradation through the ubiquitin-proteasome pathway. This process is characterized by the attachment of ubiquitin (Ub) to substrate proteins to form a poly-Ub chain, which is called ubiquitination and is mediated by three enzyme classes, including E1, E2, and E3.^1^ Deubiquitinating enzymes (DUBs) reverse ubiquitination by detaching Ub and the poly-Ub chain from substrates, thereby regulating the stability of the target substrates.^2^ Approximately 100 DUBs have been identified and categorized into seven families.^3^ DUBs are involved in various cellular pathways and are closely linked to several diseases^4^ such as cancer, inflammation, and neuronal diseases, thus prompting researchers to target DUBs for drug discovery. Indeed, one compound, called VLX1570, has been tested in phase-1 study,^5^ but it shows a rather lower selectivity to DUB species. Discovery of highly selective inhibitors will further enhance the therapeutic potential of targeting DUB.

Determination of the enzymatic activity of DUBs is useful for studying their fundamental biology and for inhibitor screening. Ubs conjugated with quenched fluorophores^6–8^ are simple and well-established probes that give a fluorescent signal after Ub cleavage. Other technologies rely on activity-based probes (ABPs), which comprise a mono-Ub recognition part conjugated with an electrophilic group at its C-terminus.^9^ These DUB ABPs capture the reacted DUBs through a covalent modification, which can be labelled by some detection groups. DUB ABPs have contributed to a better understanding of DUBs through investigations of the linkage specificity of the Ub-chain, structural determination of the DUB-Ub complex, and development of DUB inhibitors, although most of them are cell-impermeable and only applicable to cell lysates or purified proteins. Gui *et al.* have developed a cell-permeable DUB ABP by conjugating a cyclic polyarginine peptide to the N-terminus of a mono-Ub element.^10^ This technique enabled the use of intact cells for profiling DUB activity; however, the ABP-based techniques target the DUB “pool” in cells. Some studies have reported improved ABP technologies for the detection of specific types of DUB using engineered Ub variants;^11,12^ however, these require multi-step chemical syntheses for the preparation of the probes.

Here, we developed a system for monitoring selected DUB activity in living cells using genetic engineering techniques. One important challenge in this study was to control the reaction of the target enzyme independently of the endogenous DUBs. Deubiquitination occurs in cells homeostatically with very high activity, which makes it difficult to monitor the reaction of the exogenously expressed enzyme of interest. This is because the reporter group conjugated to the Ub substrate will be cotranslationally reacted to the endogenous DUBs, which is dominant over the exogenous expression of the selected DUB (Fig. 1, left panel). To overcome this, we used the bump-and-hole approach, which allows an orthogonal interaction between an engineered ligand and receptor pair.^13–15^ In this approach, the ligand is labelled with a bulky group that disrupts the binding to the wild-type receptor protein owing to steric interference. Simultaneously, the receptor protein is engineered by introducing a mutation that expands the binding site, so that the “bumped” ligand specifically occupies the “hole-modified” binding site in the receptor. With this approach, we sought to achieve a specific reaction of the selected DUB orthogonally to the cellular DUBs (Fig. 1 right panel) only by introducing point mutations to the enzyme and substrate pair. The system for orthogonal deubiquitination (ODU) is a plasmid-based approach without the need for chemical synthesis, which may provide a strategy that would complement ABP technologies.

**Fig. 1:**
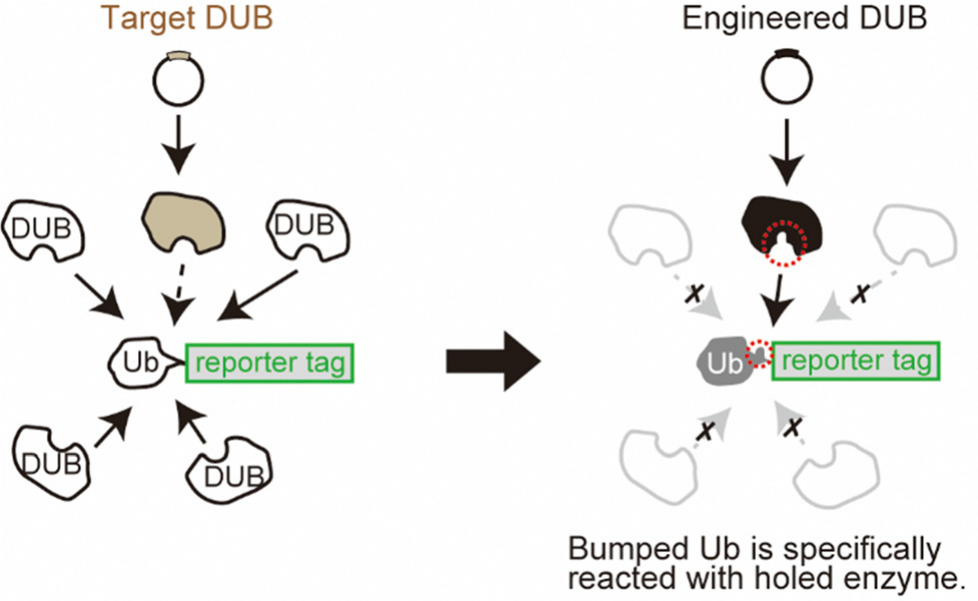
Schematic representation of the ODU system. Ub with an unmodified C-terminal sequence (white, left panel) is an excellent substrate for the pool of endogenous DUBs. The bumped Ub mutant (gray, right panel) is only targeted by “holed” DUBs (black, right panel).

As a reporter for monitoring the enzymatic reaction, we used bioluminescence resonance energy transfer (BRET). Previous trials have demonstrated that the combination of nano-luciferase (NL) as a donor and mNeonGreen (mNG) as an acceptor resulted in efficient energy transfer.^16^ We assumed that this efficient BRET will be an advantage for the high intensity of the BRET signal in the non-cleaved state. Herein, a Ub was inserted as a substrate for DUB between the donor and the acceptor. The BRET signal between NL and mNG should be decreased when the C-terminus of Ub is cleaved by the enzyme of interest (Fig. 2A). We introduced some mutations to the Ub: first, all the Lys residues were substituted to Arg to avoid a poly-Ub chain formation, and thus extra degradation of the probe. This Lys-free Ub mutant was called UbK0 and described as Ub* in this article. Second, G75 in the G75–G76 motif of Ub*, which can be recognized by the catalytic site of DUBs and is a highly conserved sequence among species,^17^ was substituted to prevent the enzymatic activity of endogenous DUB. This substitution was to another amino acid with a bulkier side chain that blocks the interaction with the catalytic site of wild-type DUB.^18^ We envisioned that a G75 mutant will be a bumped substrate that can be targeted by an engineered mutant enzyme.

**Fig. 2:**
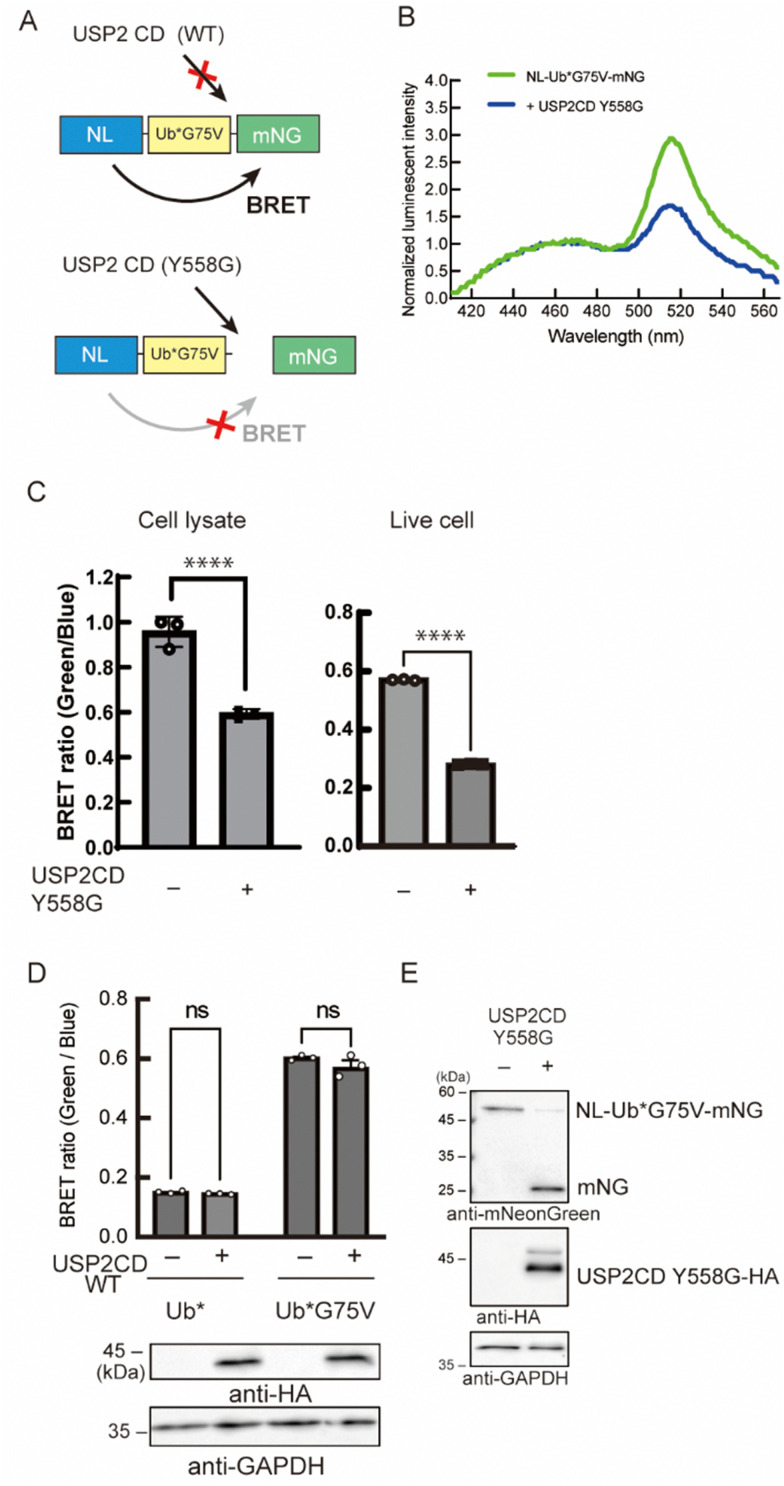
Establishment of the ODU system. (A) Specific reaction of the BRET probe harboring the Ub*G75V mutant with the USP2 catalytic domain (CD) mutant. NL, Nano-luciferase. mNG, mNeonGreen. (B) Luminescence emission spectra of NIH3T3 cells expressing the BRET-G75V probe with or without the USP2CD Y558G mutant. (C) Emission ratio of NIH3T3 cells expressing the BRET-G75V probe with or without the USP2CD Y558G mutant. The data represent the mean ± SD of three independent experiments. Statistical analysis was conducted using Student's *t*-test. ****p < 0.0001. *n* = 3. (D) Emission ratio of NIH3T3 cells expressing the BRET-G75V probe with or without HA-tagged wild-type USP2 CD. Ns, not significant. *n* = 3. Expression of HA-tagged wild-type USP2 CD was confirmed by immunoblotting using an antibody against a HA tag. GAPDH was used for loading control. (E) Immunoblotting analysis of cell lysates of NIH3T3 cells expressing the BRET-G75V probe with or without the HA-tagged USP2CD Y558G mutant using antibodies against mNeonGreen, HA tag, or GAPDH. GAPDH was used as a loading control.

Initially, we examined the reactivity of G75 mutants to endogenous DUBs by measuring the BRET signal (Fig. S1A, ESI†). A fusion protein, comprising NL, Ub*, and mNG, was stably expressed in NIH3T3 cells, and the BRET signal was quantified. A low BRET ratio was observed in cells expressing the probe harboring the Ub* with an unmodified C-terminal sequence. The signal was recovered when we replaced G75 with Val. We further confirmed the reactivity of these probes toward endogenous DUBs using immunoblotting (Fig. S1B, ESI†). These results can be explained by the bulkier side chain of Val that blocked the access to the reaction center of DUB. The G75A mutant showed the same tendency as the wild-type, whereas the G75S/C mutants showed a partial increase in BRET signals. The other 16 mutants behaved as resistant mutants toward DUB cleavage like the Val mutant. These reactivities of the G75 mutants were identical to our previous results.^19^ Because the G75V mutant has been characterized by previous studies,^18,20^ it was chosen as the bumped substrate.

Next, we engineered the enzyme for the selective cleavage of the bumped Ub substrate. In the catalytic center of many DUBs, aromatic amino acids like Tyr, Phe, or Trp, are highly conserved and interact with the C-terminal region of Ub, especially the G75 residue (Fig. S2A and B, ESI†).^17,21^ These result suggest that the Tyr residue acts as a gatekeeper to prevent larger side chains from penetrating into the catalytic core. We envisioned that the replacement of this aromatic gatekeeper residue to an amino acid with a smaller side chain will generate a hole that would accommodate a modified Ub substrate with a bumped penultimate residue. This hole-modified enzyme should cleave the C-terminus of Ub*G75V of the probe, resulting in the decrease of the BRET signal (Fig. 2A). Using ubiquitin-specific protease 2 (USP2) as a model enzyme, we prepared a vector encoding a catalytic domain (CD) of USP2 (N259-M605) in which Y558 was mutated to Gly. The engineered enzyme was co-expressed with NL-Ub*G75V-mNG (BRET-G75V probe) in NIH3T3 cells, and the BRET ratio was measured. We found that the BRET ratio of the probe was decreased in the presence of the USP2CD Y558G mutant (Fig. 2B). The decreased BRET ratio was observed in cell lysates and in living cells (Fig. 2C). In contrast, the BRET ratio was not changed in cells expressing the wild-type USP2CD (Fig. 2D). Next, we examined the BRET-G75V probe reactivity to the engineered USP2CD by immunoblotting. In the absence of engineered USP2CD, a band around 50 kDa was detected as a fusion protein of NL-Ub*G75V-mNG (Fig. 2E), suggesting that the probe was not cleaved by the endogenous wild-type DUBs. In contrast, the fusion protein band almost disappeared in the presence of the engineered USP2CD, and the liberated mNG was detected.

We then sought to develop a simultaneous expression system for the BRET-G75V probe and the modified enzyme because the enzymatic reaction efficiency is closely related to the abundance balance between the substrate and enzyme. In particular, low transfection efficiency of an enzyme-encoding plasmid affects the reaction efficiency. To this end, we constructed bicistronic plasmids that enable coexpression of the two components under the 2A peptide or internal ribosome entry site (IRES) (Fig. S3A, ESI†). The gene encoding the enzyme was connected upstream of the P2A or IRES, either of which controls the expression of the BRET-G75V probe (Enzyme-P2A-Probe or Enzyme-IRES-Probe). We observed a significant decrease in BRET signal in the presence of the engineered USP2CD in either system compared with the control and wild-type enzyme (Fig. S3B and C, ESI†). The dynamic BRET range was slightly affected when the gene order was swapped (Probe-P2A-Enzyme or Probe-IRES-Enzyme) (Fig. S4, ESI†). Given that the expression levels of the elements downstream of IRES and P2A are generally lower than those upstream,^22,23^ these results suggest that a higher expression of the modified enzyme is preferable for the cleavage reaction of Ub*G75V.

Next, we tested whether the ODU system is applicable to DUBs other than USP2. Among several USP family members, we observed a decrease in BRET in the presence of engineered USP15. USP15 is involved in various processes including immune and inflammatory processes of leukocytes^24^ and in regulation of RNA processing in neuronal cells.^25^ USP15 has a catalytic core similar to other DUBs and shows a broad range of reactivity to many Ub linkage types.^26^ Y892 is in the catalytic site of USP15 and is thought to act as a gatekeeper residue like Y558 of USP2. We introduced a Y892G mutation to enlarge the catalytic core and examined the engineered full-length USP15 with a bumped Ub*. The spectrum and quantification results showed that the BRET ratio was significantly decreased in the presence of the HA-tagged USP15 mutant (Fig. 3A and B), suggesting that the ODU system can be applied to USP15.

**Fig. 3:**
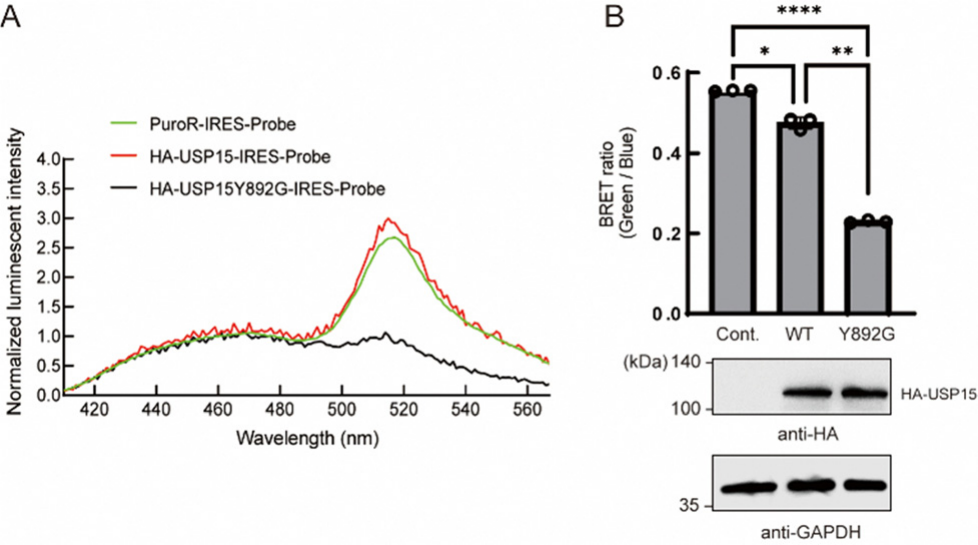
Application of the ODU system for USP15. (A) Luminescence emission spectra of NIH3T3 cells expressing the BRET-G75V probe with wild-type or Y892G USP15. The PuroR-IRES-Probe plasmid was used as a control. (B) Emission ratio of intact NIH3T3 cells expressing the BRET-G75V probe with wild-type or Y863G USP15. The PuroR-IRES-Probe plasmid was used as a control. The data represent the mean ± SD of three independent experiments. Statistical analysis was conducted using one-way ANOVA analysis (Tukey's test). *p < 0.05, **p < 0.01, ****p < 0.0001. *n* = 3. (C) Immunoblotting analysis of cell lysates of NIH3T3 cells expressing the BRET-G75V probe with or without the HA-tagged USP15 Y863G mutant using anti-HA or anti-GAPDH antibodies. GAPDH was used as a loading control.

Finally, we performed a cell-free assay and bioinformatic analyses to characterize the reactivity of the engineered enzyme and Ub. We made the wild-type and G75V ubiquitins, which have a Flag tag and biotin ligase recognition sequence (bls) at their N- and C-terminal sites, respectively, as previously reported with slight modifications.^27^ It has been shown that the Flag tag and bls peptides did not disrupt the enzymatic activity of the DUB *in vitro* assay system. It allows us to distinguish the enzymatic reaction by observing a band-shift after DUB-cleavage of 14 amino acids from the C-terminal site. We also prepared wild-type and Y892G mutant recombinant USP15 proteins based on the procedure described previously.^28^ As shown in Fig. 4A, the wild-type USP15 did not react with UbG75V, while the Y892G mutant cleaved the bls sequences at the C-terminus of Ub in both the wild-type and G75V mutant. To verify the results of the *in vitro* assay, we performed bioinformatic analyses. As the structure of the USP15 catalytic center (C298) recognizing the C-terminus of Ub has not yet been determined, the structure of USP15 in complex with Ub was predicted by AlphaFold2^29,30^ (Fig. S5, ESI†). Modelling was performed with the wild-type of USP15 (residues 284–948) and the wild-type of Ub (full length). The predicted model with the highest pLDDT score is shown in Fig. S5A and B (ESI†), and the predicted aligned error (PAE) plot for the model is shown in Fig. S5C (ESI†). AlphaFold2 predicted that the catalytic center of USP15 (C298) recognized the C-terminus of Ub, and the pLDDT score and PAE plot showed that the structure around G75 of Ub was highly accurate. We further introduced the mutation on G75 of Ub and Y892 of USP15 and then calculated the interaction energies of each complex by program FoldX^31,32^ (Fig. 4B). This prediction showed that the G75V mutation of Ub caused steric hindrance to Y892 of USP15, increasing the interaction energy of the complex by 9.4 kDa mol^−1^. Since lower values of interaction energy indicate a more favourable interaction, the G75V mutation of Ub inhibits the binding of USP15 to Ub. On the other hand, the introduction of a Y892G mutation in USP15 in addition to G75V in Ub eliminated the steric hindrance and improved the interaction energy to almost the same level as the wild-type complex. These results supported our observation of cell experiments.

**Fig. 4:**
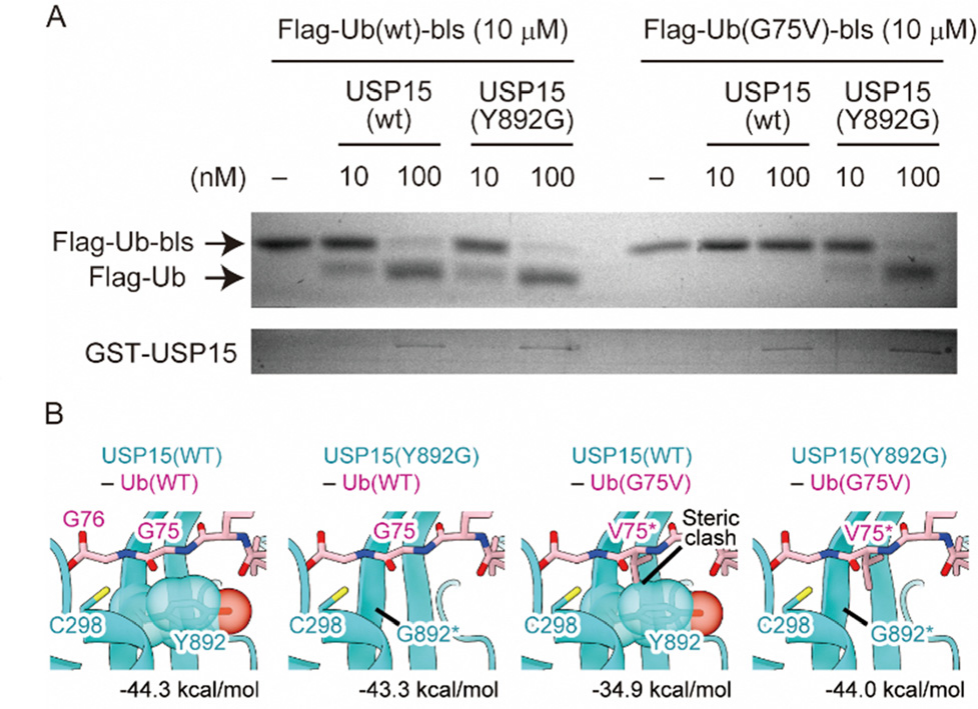
Characterization of reactivity between engineered Ub and USP15. (A) The reaction mixture of recombinant proteins of Ub and USP15 was separated by SDS-PAGE, and the resultant gel was stained with Coomassie Brilliant Blue. (B) Close-up views of the area around Gly75 of Ub in the predicted model for the complex between USP15 and Ub. Combinations of WT or mutant for USP15 and Ub are shown above each panel. Interaction energies of each complex calculated by the program FoldX (version 5.0) are shown below each panel. USP15 and Ub are cyan and pink, respectively. The side chain of Tyr892 in USP15 is shown as a translucent sphere model. Asterisks indicate the mutated residues.

The ODU system is based on the specific reaction between an engineered DUB and a Ub substrate. The engineered DUB can cleave the peptide bond between the C-terminus of Ub and the N-terminus of mNG. This linkage is thought to be a surrogate of the N-terminal ubiquitination of substrate proteins, which could be targeted by the model two enzymes used in this study. There have been several DUBs that show a similar preference for the Ub-linkage (*e.g.* USP5, 16, 21, 24, 36, 38, CYLD, OTULIN).^28,33^ Further applications to the other DUBs are expected. In contrast, Ub also has various linkage patterns on the basis of the isopeptide bond between the C-terminus of Ub and the Lys residues of another Ub or the substrates.^34^ We note that it may be difficult to apply our system to certain types of DUBs, which show a strict preference to side-chain Ub linkage. We expect further technical advances on genetic engineering regarding the selective creation of linkage specific Ub chains in living cells, which may expand the availability of our system.

It should be noted that the hole USP15 mutant could cleave not only the bump-Ub mutant but also wild-type Ub. This suggests that bump Ub may compete with wild-type Ub, including endogenous Ub molecules, for access to the hole USP mutant. However, we emphasize that wild-type Ub can also react with abundant native DUB families, which is likely dominant over the exogenous hole USP mutant. The effect of the hole-USP mutant on the endogenous Ub machinery should be considered in some cases.

In conclusion, we developed a genetic-based strategy, called ODU, to determine the deubiquitinating activity of a selected DUB in living cells. The system is based on the point mutation of the key residues in both the substrate and enzymes. The aromatic gatekeeper residues, including Tyr, Phe, and Trp, are adjacent to the catalytic His residue and are highly conserved among all the DUB family (Fig. S2A, ESI†), suggesting that our system may be potentially applicable to various types of DUBs. This may be useful for investigating target enzymes with aspects of post-translational modifications, localization, and specific cellular environments such as redox states.^35^ Furthermore, the bump-and-hole approach has been originally used to elucidate the substrate protein of the target enzyme.^13,15^ We expect that our tool can also help identify the unknown substrate of DUBs.

This work was supported by MEXT/JSPS KAKENHI Grant Number JP 21H00271 (Y. M.), 21H00285 (S. Y.), 21H00287 (H. T.), 22J13230 (Y. U.), 21H00283 (Y. S.), and 21H02418 (Y. S.), and MEXT Leading initiative for Excellent Young Researchers (Y. S.), and Tottori University Research Support Project for the Next Generation (Y. S.) and Takeda Science Foundation (Y. M.). We thank the Alliance for Research on the Mediterranean and North Africa, University of Tsukuba, for the use of facilities. We thank Ms Tomoko Nakagawa and Ms Chie Furukawa for technical assistance on preparation of recombinant proteins.

## Conflicts of interest

There are no conflicts to declare.

## Supplementary Material

CB-004-D3CB00095H-s001
