# Supplementary material for: A strategy for orthogonal deubiquitination using a bump-and-hole approach
Source: RSC Chem Biol. 2023 Oct 2;4(11):879–83. doi: 10.1039/d3cb00095h (PMC10619139; doi:10.1039/d3cb00095h)
Supplement: CB-004-D3CB00095H-s001 [file CB-004-D3CB00095H-s001.pdf]

Electronic Supplementary Material (ESI) for RSC Chemical Biology.
This journal is © The Royal Society of Chemistry 2023

Supplementary Information

A strategy for orthogonal deubiquitination using a bump-and-hole approach

Takumi Suzuki[a], Yuki Utsugi[a], Satoshi Yamanaka[b], Hirotaka Takahashi[b], Yusuke Sato[c], Tatsuya Sawasaki[b], Yusaku Miyamae[d]*

[a]*Master's/Doctoral Program in Life Science Innovation, Graduate School of Comprehensive Human Sciences, University of Tsukuba, Tsukuba, Ibaraki, 305-8572, Japan*
[b]*Division of Cell-Free Science, Proteo-Science Center, Ehime University, Matsuyama, Ehime, 790-8577, Japan*
[c]*Center for Research on Green Sustainable Chemistry, Tottori University, Tottori, 680-8552, Japan*
[d]*Institute of Life and Environmental Sciences, University of Tsukuba, Tsukuba, Ibaraki, 305-8572, Japan*

*Corresponding author: Yusaku Miyamae
E-mail address: miyamae.yusaku.fw@u.tsukuba.ac.jp (Y. Miyamae)
Tel.: +81-29-853-6417 (Y. Miyamae)

Materials and Methods

Plasmids

The genes of interest were cloned into retroviral pBMN vectors. The cDNA sequences used were: mNeonGreen-DEVD-NanoLuc (Addgene plasmid #98287), Ub(K0) (*Homo sapien* Ubiquitin-K0, Addgene plasmid #17603), USP2 (*Homo sapien*, RIKEN BRC plasmid #IRAL008G18), and USP15 (*Homo sapien*, Addgene plasmid #22570). Plasmid vectors were constructed by restriction enzyme cloning or Seamless ligation cloning extract (SLiCE) [1].

Cell culture

Mouse fibroblast-like cell line NIH3T3 (RIKEN BRC, Japan, #RCB1862) and Phoenix Ecotropic packaging cell line (kindly gifted by Prof. Ohneda, University of Tsukuba) were cultured in Dulbecco's modified eagle medium (DMEM) (Sigma–Aldrich, #D5796) supplemented with 10% heat-inactivated fetal bovine serum (Gibco, #10270-106), 10,000 U/mL penicillin, and 10 μg/mL streptomycin (LONZA, #17-602E). Cells were maintained at 37°C and 5% CO_2 in a humid atmosphere.

Transfection and Transduction

Retroviruses were produced by transfecting Phoenix Ecotropic packaging cell line with pBMN plasmids using a standard TransIT-LT1 (Mirus, #MIR-2300) protocol. Supernatants containing viruses were harvested 48 h post-transfection and filtered with a 0.45 μm filter. Cells were incubated with the viral supernatants supplemented with polybrene (Nacalai Tesque, Inc., #12996-81) for 4 h at 37°C then cultured in growth media for 48 h to allow for viral integration.

BRET signal detection

Cells were washed with PBS, and then phenol red-free, high glucose DMEM (Nacalai Tesque, Inc., #08489-45) was added to each well. BRET signal was measured using the Nano-Glo Luciferase Assay System (Promega, #N1110) or the Nano-Glo Live Cell Assay System (Promega, #N2011) according to the manufacturer's instructions, and detected by Varioskan LUX (Thermo Fisher Scientific, #VL0L00D2) equipped with luminescent filters (460/80 nm, Thermo Fisher Scientific, #F460/80; 515/30 nm, Thermo Fisher Scientific, #F51530).

Immunoblotting and antibodies

Cells were washed twice with PBS and lysed with a radio-immunoprecipitation assay buffer (Sigma-Aldrich, #R0278). Lysates were resolved by SDS-PAGE and transferred to a PVDF membrane (Merck Millipore, #IPFL00010). The antibodies used: anti-mNeonGreen (rabbit, #53061S, Cell Signaling Technology), anti-HA (rabbit, #3724S, Cell Signaling Technology), and anti-GAPDH (mouse, MA5-15738, Thermo Fisher Scientific). Horseradish peroxidase (HRP)-conjugated anti-rabbit (Cell Signaling Technology, #7074) or anti-mouse secondary antibodies (Cell Signaling Technology, #7076) were used at a 1:3000 dilution. Immunoreactive bands were visualized using immobilon western chemiluminescent HRP substrate (Merck Millipore, #WBKLS0500) and the Odyssey Fc Imaging System (LI-COR).

Preparation of purified GST-USP15

Full-length ORF region of USP15 was subcloned into pEU-E01-GST-TEV-MCS vector (pEU-GST, CellFree Sciences). Based on the pEU-GST-USP15 construct, the amino acid substitutions of tyrosine to glycine (Y892 for USP15) were introduced using PrimeSTAR mutagenesis basal kit (Takara Bio). The wheat-cell free expression and purification of GST-tagged USP15 were performed based on the procedure described previously with slight modifications[2]. The cell-free translation reaction was done with a wheat germ extract for GST-affinity purification (WEPRO1240G, CellFree Sciences). 3.6 mL of each cell-free translation product was mixed with 150 mM NaCl and 10 mM DTT, and GST-tagged USP15 were captured with Glutathione Sepharose 4B beads (GE Healthcare). Then, the beads were washed three times with PBS and GST-tagged USP15 were eluted with 200 mL of elution buffer (50 mM Tris-HCl, pH 8.0, 50 mM NaCl, 10 mM reduced glutathione). The concentration of each purified protein was determined by bovine serum albumin (BSA) standard.

Preparation of purified Flag-Ub-bls

The recombinant proteins of Flag-Ub (wt or G75V)-biotin ligase recognition site (bls; LNDIFEAQKIEWHE) were prepared as previously reported with slight modifications[3]. The ORF of human ubiquitin (wt or G75V mutant) was fused with the nucleotide sequences of Flag-tag and bls at the 5’- and 3’-portion, respectively. The whole nucleotide sequence was verified, and they were ligated into a modified pET16b plasmid from which the hexa-histidine tag sequence was removed (Addgene). The recombinant proteins of Flag-Ub (wt or G75V)-bls were expressed in *E. coli* BL21 (New England Biolabs), and induced by 0.5 mM isopropyl-β-D-thiogalactopyranoside at 37°C for 6 h. The pellet of *E. coli* was homogenized by sonication in TBS buffer, and centrifuged at

10,000 rpm for 10 min. The supernatant was heated at 70°C for 15 min to denature proteins from *E.coli*, and centrifuged again. The supernatants were loaded onto a HiLoad 16/60 Superdex75 column (Cytiva) in Tris-HCl buffer (pH 7.5) containing 50 mM NaCl and 5 mM β-mercaptoethanol. The fractions abundant in the Flag-Ub(wt or G75V)-bls were purified by HiLoad 16/60 Superdex75 column again to obtain higher purity samples. Purified samples were concentrated to ~5 g/L with an Amicon Ultra-4 3,000 MWCO filter (Millipore) according to the manufacturer's instructions and flash-frozen in liquid nitrogen and stored at -80°C until use.

In vitro digestion assay

Flag-Ub (wt or G75V)-bls (10 μM) and GST-USP15 (wt or Y892G) were dissolved in 20 μL buffer containing 50 mM Tris-HCl (pH7.5), 5 mM DTT and incubated at 37°C for 90 min. 10 μL of the reaction mixture was resolved by SDS-PAGE (15% gel) and the resultant gel was stained with Coomassie Brilliant Blue (Nacalai Tesque, Inc., #09408-52).

Calculation of interaction energy for predicted models

To predict the structure, we used the ColabFold (v1.5.2) Google Colab notebook called AlphaFold2_mmseqs2 [4, 5]. Modelling was performed with the wild type of USP15 (residues 284-948) and the wild type of Ub (full length). The predicted model with the highest pLDDT score is shown in Fig. S5A and B, and the Predicted Aligned Error (PAE) plot for the model is shown in Fig. S5C. The predicted model was further optimized using the <Repair object> command of the program FoldX (version 5.0) [6, 7]. We then introduced each mutation with the <Mutate residue> command of the program FoldX. Finally, the interaction energy between USP15 and Ub of each model was analyzed through the <Interaction energy of molecules> command of the program FoldX.

Statistical analysis

All statistical analyses were performed using GraphPad Prism9. The methods and representative symbols are described in the figure legends. Symbols mean significant differences compared with the mean values of the indicated number of independent experiments. A paired Student's *t*-test or one-way ANOVA was used to compare between two or more experimental groups, respectively. $p < 0.05$ was considered statistically significant.

References

1. K. Motohashi, *BMC Biotechnol*, 2015, **15**, 47.

2. H. Takahashi, S. Yamanaka, S. Kuwada, K. Higaki, K. Kido, Y. Sato, S. Fukai, F. Tokunaga and T. Sawasaki, *Biomedicines*, 2020, **8**.
3. S. Yamanaka, Y. Sato, D. Oikawa, E. Goto, S. Fukai, F. Tokunaga, H. Takahashi and T. Sawasaki, *Biochem Biophys Res Commun*, 2020, **524**, 1-7.
4. J. Jumper, R. Evans, A. Pritzel, T. Green, M. Figurnov, O. Ronneberger, K. Tunyasuvunakool, R. Bates, A. Žídek, A. Potapenko, A. Bridgland, C. Meyer, S. A. A. Kohl, A. J. Ballard, A. Cowie, B. Romera-Paredes, S. Nikolov, R. Jain, J. Adler, T. Back, S. Petersen, D. Reiman, E. Clancy, M. Zielinski, M. Steinegger, M. Pacholska, T. Berghammer, S. Bodenstein, D. Silver, O. Vinyals, A. W. Senior, K. Kavukcuoglu, P. Kohli and D. Hassabis, *Nature*, 2021, **596**, 583-589.
5. M. Mirdita, K. Schütze, Y. Moriwaki, L. Heo, S. Ovchinnikov and M. Steinegger, *Nat Methods*, 2022, **19**, 679-682.
6. J. Schymkowitz, J. Borg, F. Stricher, R. Nys, F. Rousseau and L. Serrano, *Nucleic Acids Res*, 2005, **33**, W382-388.
7. J. Van Durme, J. Delgado, F. Stricher, L. Serrano, J. Schymkowitz and F. Rousseau, *Bioinformatics*, 2011, **27**, 1711-1712.

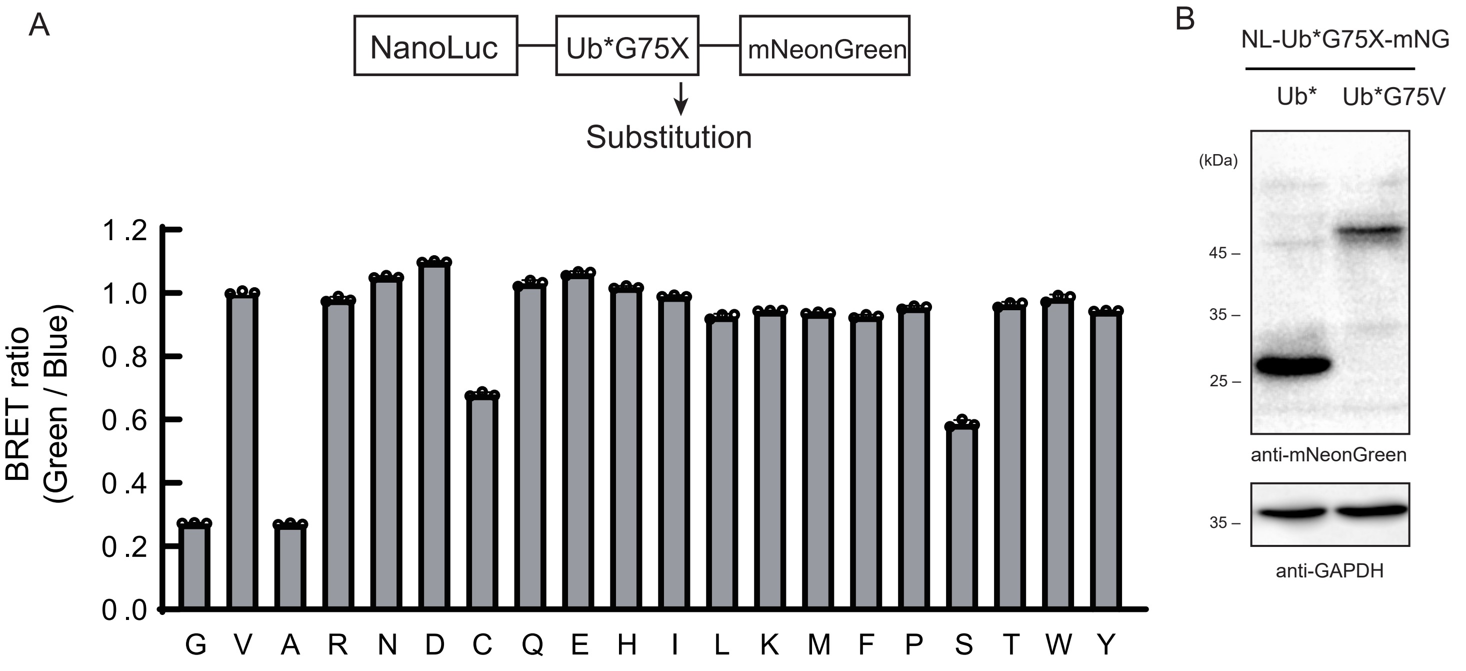

Fig. S1 Reactivity of Ub*G75 mutants to endogenous DUBs.

(A) NIH3T3 cells were transfected with the plasmids encoding NL-Ub*G75X-mNG. Emission ratio of the lysates prepared from the transfected cells was displayed. n = 3.

(B) Immunoblotting analysis of cell lysates of NIH3T3 cells expressing the NL-Ub*-mNG or NL-Ub*G75V-mNG using antibodies against mNeonGreen or GAPDH. GAPDH was used as a loading control.

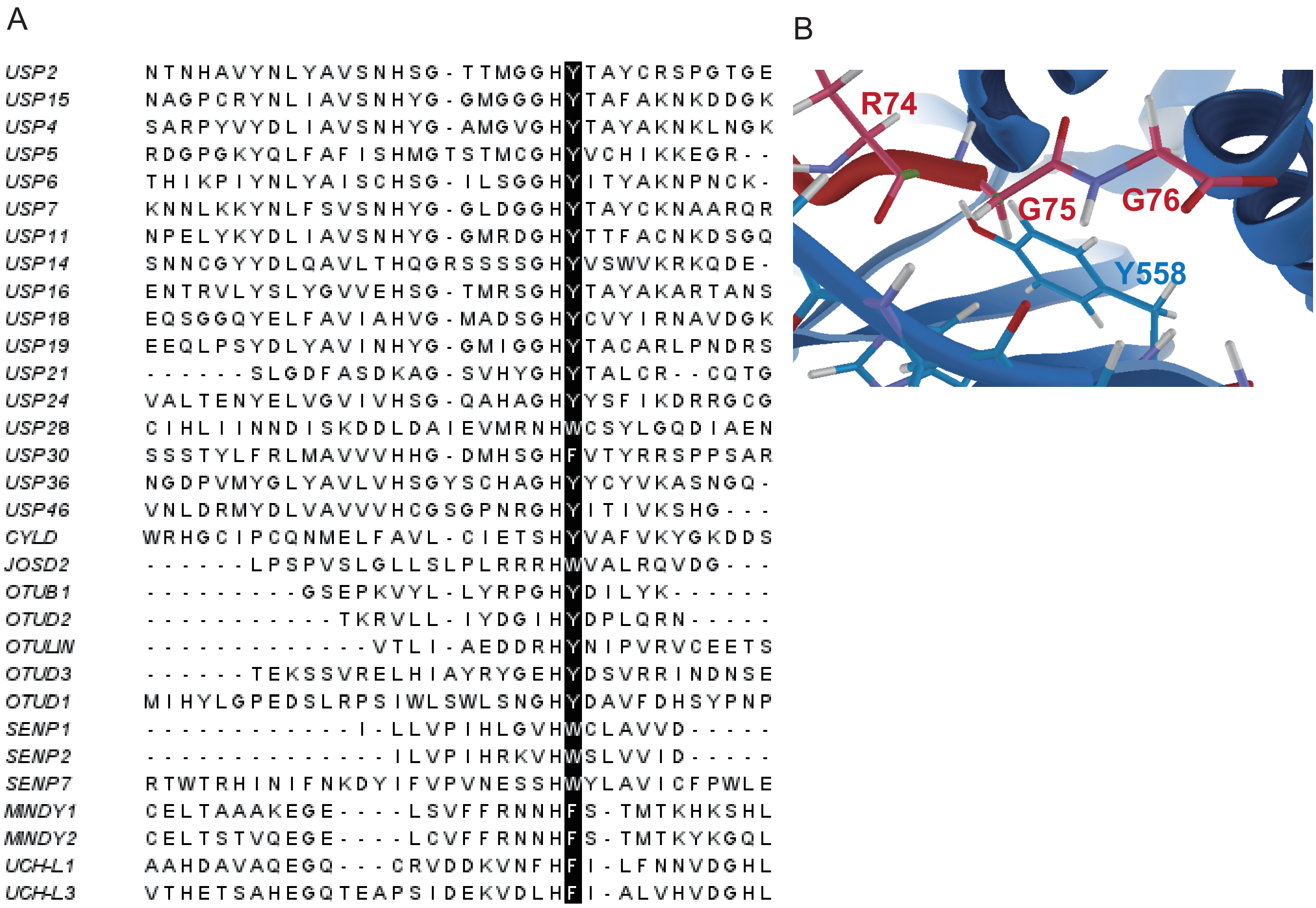

Fig. S2 Role of aromatic amino acid as the gatekeeper residue in DUBs.

(A) Sequence alignment of representative DUBs. The sequences of catgalytic core region of representative DUBs were compared. The conserved aromatic gatekeeper residues are indicated by black background. (B) Recognition of G75-G76 motif of Ub by the catalytic core in USP2 (PDB code: 2HD5). C-terminal residues of Ub and catalytic core of USP2 were displayed in red and blue, respectively.

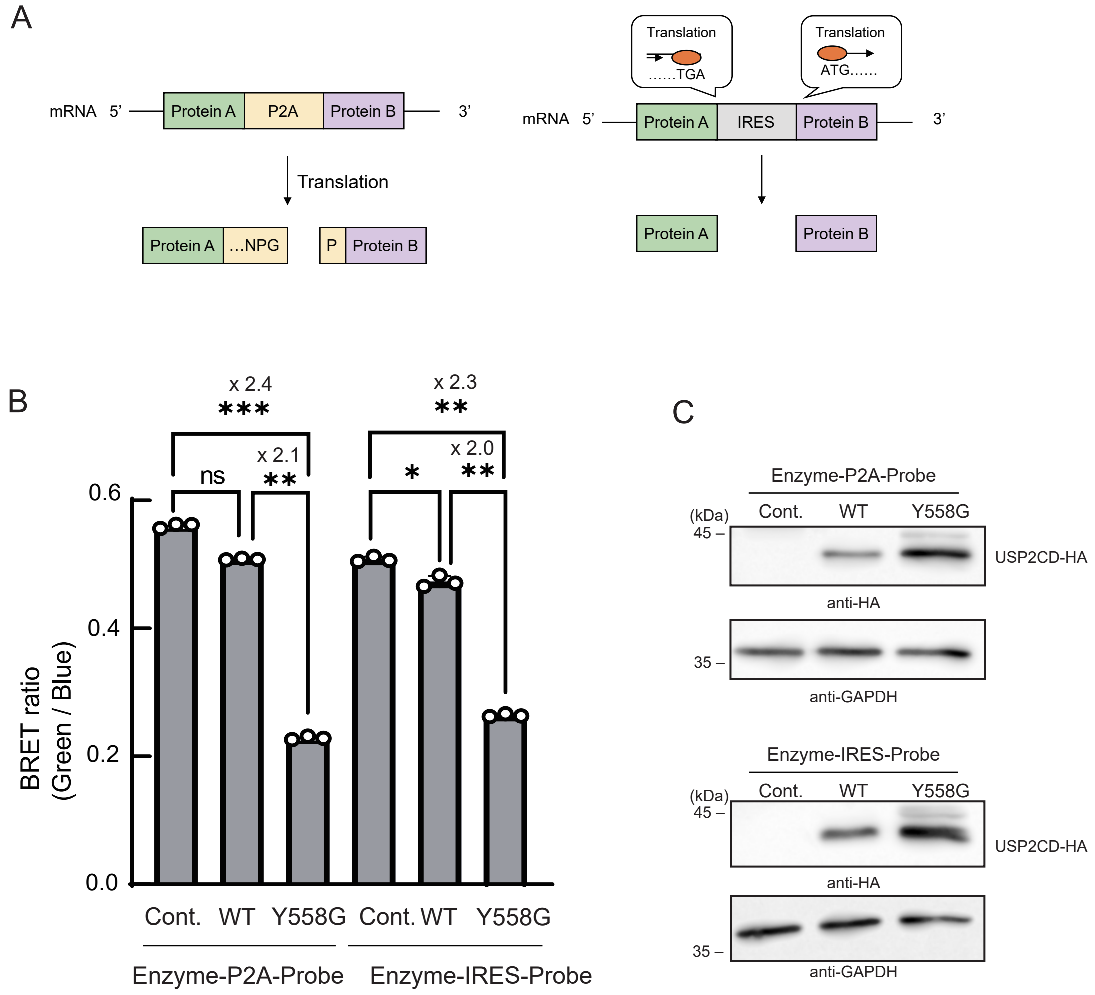

Fig. S3 Optimization of the ODU system expression method.

(A) Schematic of translation under P2A and IRES. (B) NIH3T3 cells were transduced with bicistronic plasmids encoding wild type or Y558G HA-tagged USP2CD (enzyme) and the BRET-G75V probe (probe). Cont. means no enzyme-encoding gene in the indicated insertion site. BRET signals from intact cells were measured. The data represent the mean ± SD of three independent experiments. Statistical analysis was conducted using one-way ANOVA analysis (Tukey' s test). *p < 0.05, **p < 0.01, ***p < 0.001. n = 3. (C) Immunoblotting analysis of cell lysates of NIH3T3 cells expressing the Enzyme-IRES-Probe or Enzyme-P2A-Probe plasmid using anti-HA or anti-GAPDH antibodies. GAPDH was used as a loading control.

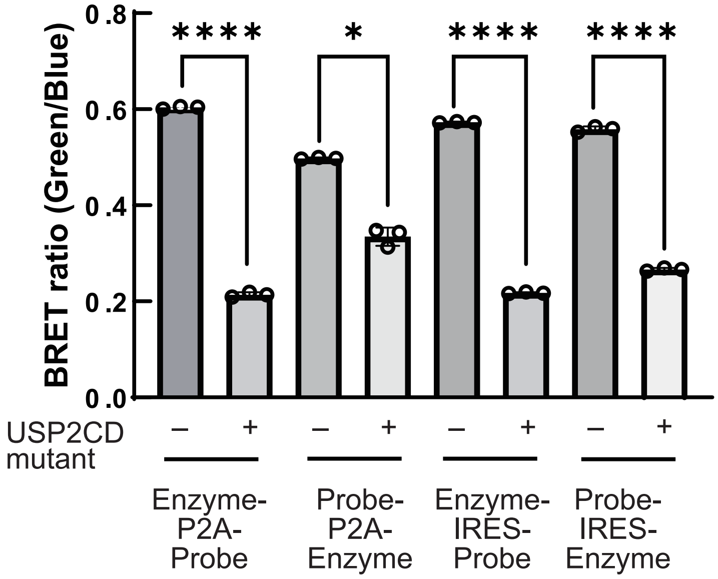

Fig. S4 Comparison for expression method for ODU system.

Emission ratio of the intact NIH3T3 cells expressing the bicistronic vectors encoding HA-tagged USP2CD Y558G mutant (Enzyme) and BRET-G75V probe (Probe). - means no genes encoding Enzyme in the indicated insertion site. The data represent the mean ± SD of results from three independent experiments.Statistical analysis was conducted using one-way ANOVA (Tukey’ s test). *$p < 0.05$, ****$p < 0.0001$. $n = 3$.

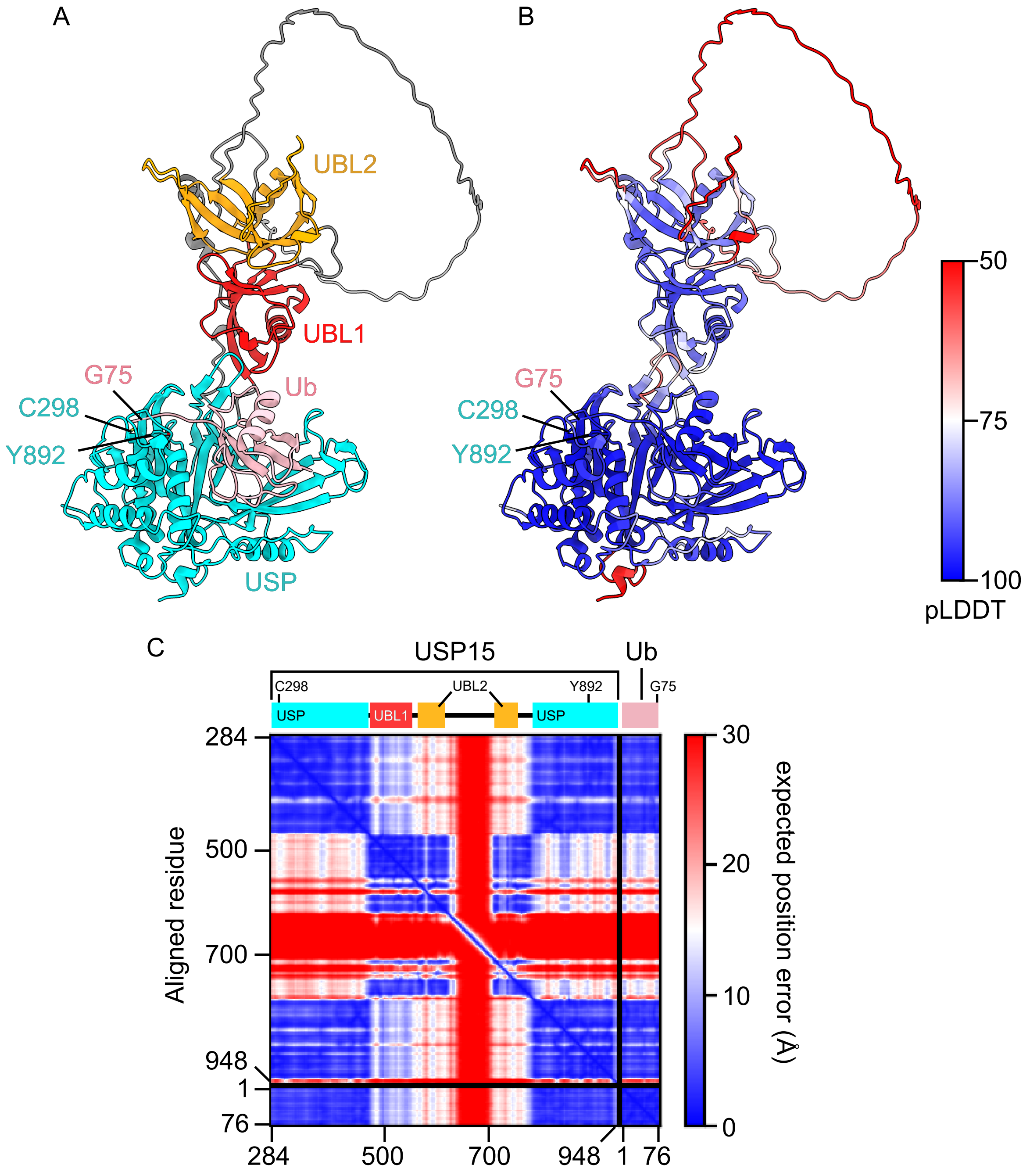

Fig. S5 AlphaFold2 model of the catalytic region of USP15 in complex with Ub.

(A) AlphaFold2 predicted that two UBL domains (UBL1 and UBL2) are inserted into the USP domain of the catalytic region of USP15 (residues 284-948). USP, UBL1, and UBL2 domains of USP15 are colored cyan, red, and orange, respectively. Ub is colored pink. (B) The AlphaFold2 model is colored with a red-to-blue gradient according to the structure prediction confidence as estimated by the predicted Local Distance Difference Test (pLDDT) (red is low confidence and blue is high confidence). (C) Predicted Aligned Error (PAE) plot for the AlphaFold2 model. Protein boundaries are indicated by black lines in the PAE plot. The plot is colored with a red-to-blue gradient according to the expected position error (Å) (red is low confidence and blue is high confidence). The domain composition of USP15 is shown above the plot.

Supplementary full gel and blots

Fig. 2D

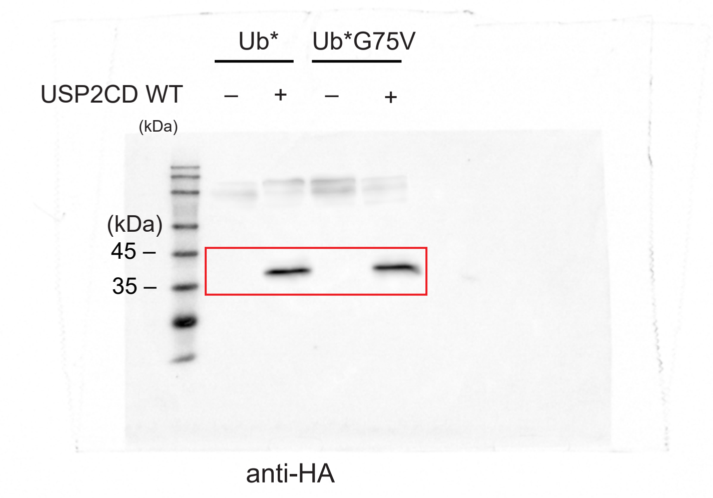

Fig. 2D

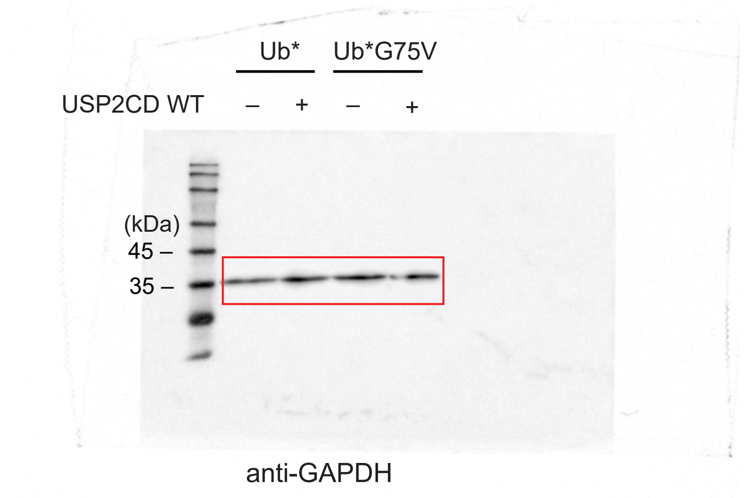

Fig. 2E

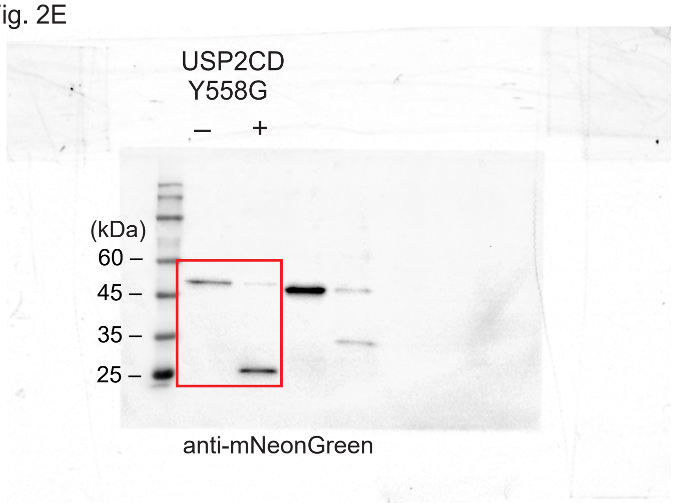

Fig. 2E

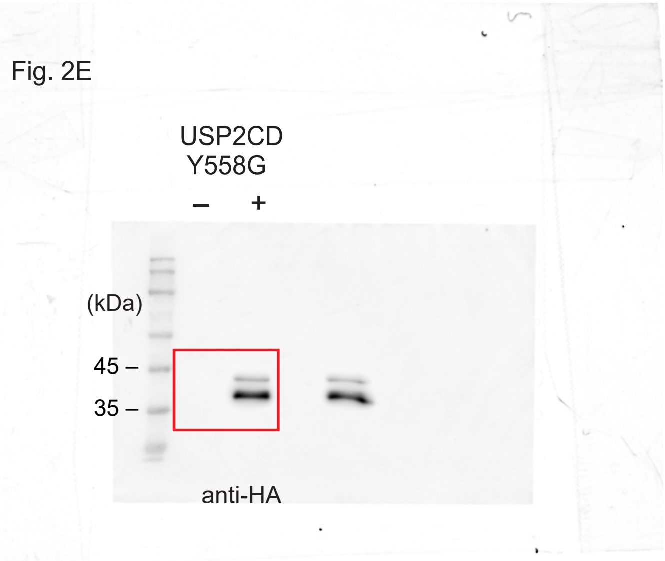

Fig. 2E

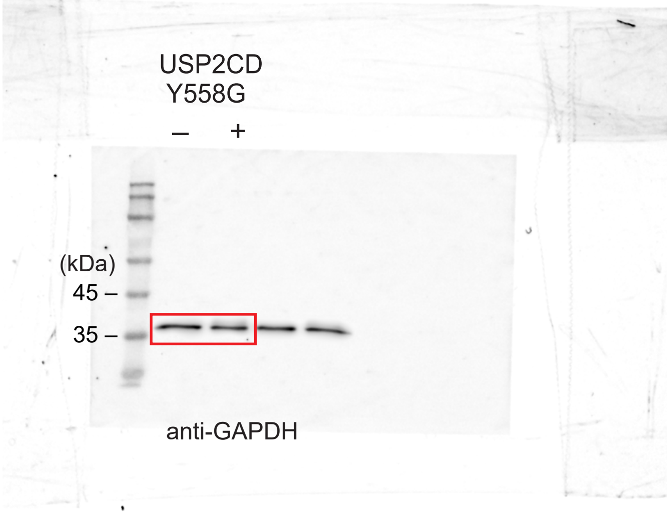

Full western blots and gels in the manuscript.

Supplementary full gel and blots

Fig. 3B

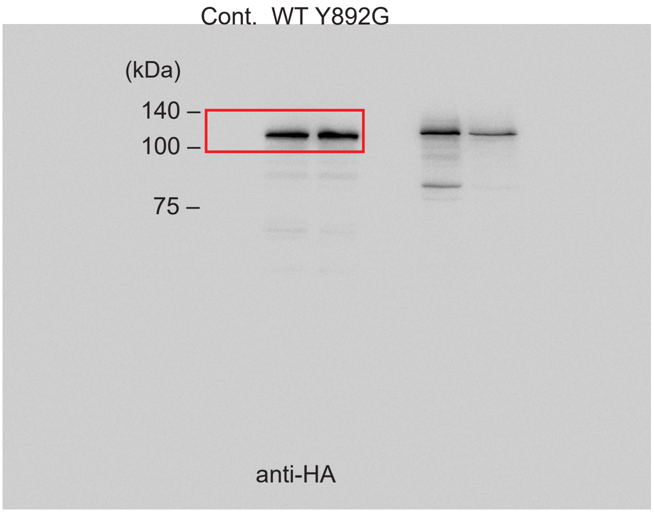

Fig. 3B

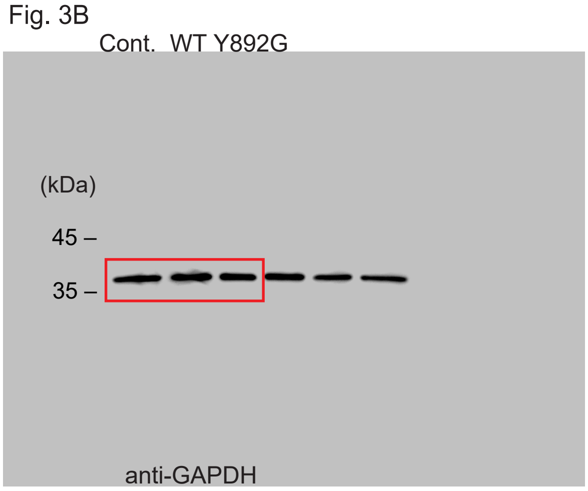

Fig. 3B

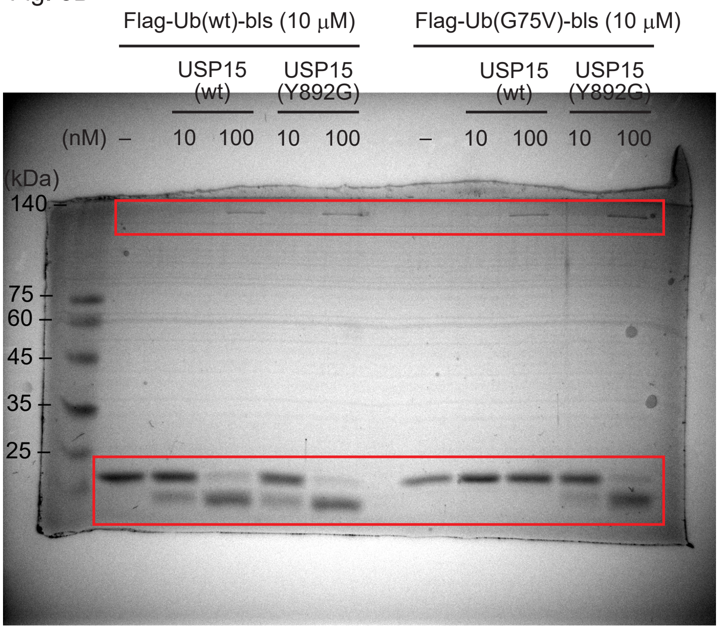

CBB staining

Full western blots and gels in the manuscript.

Supplementary full blots

Fig. S1

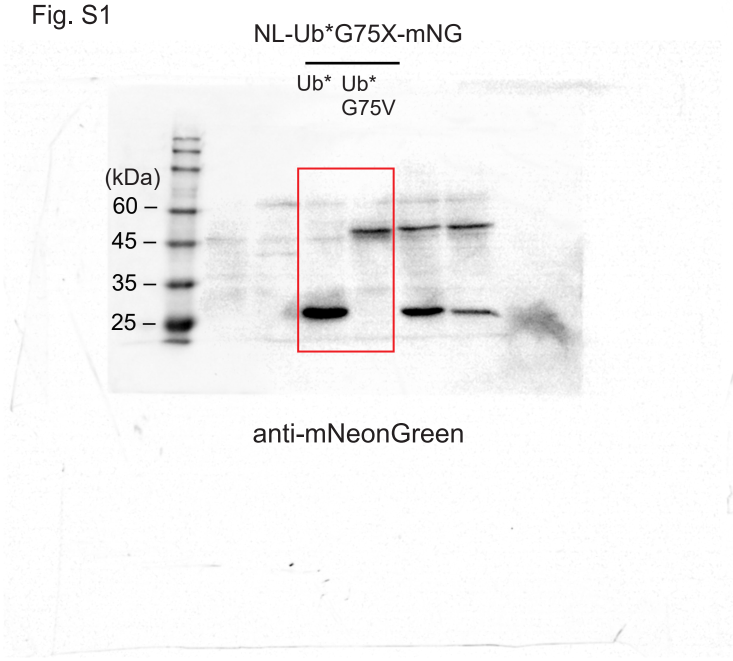

Fig. S1

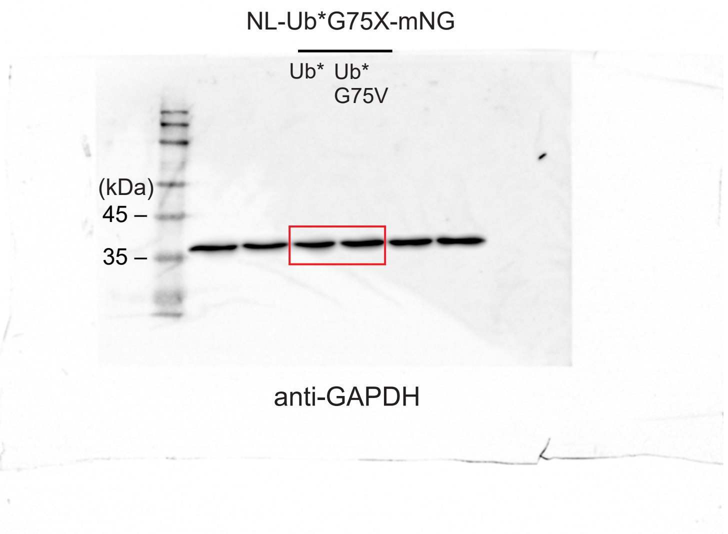

Fig. S3

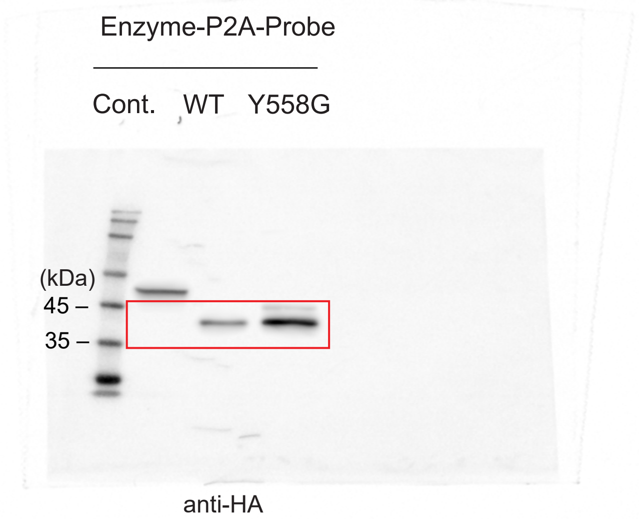

Fig. S3

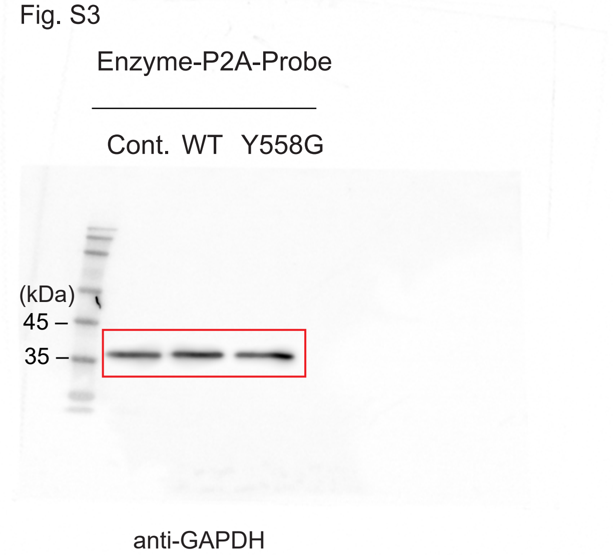

Fig. S3

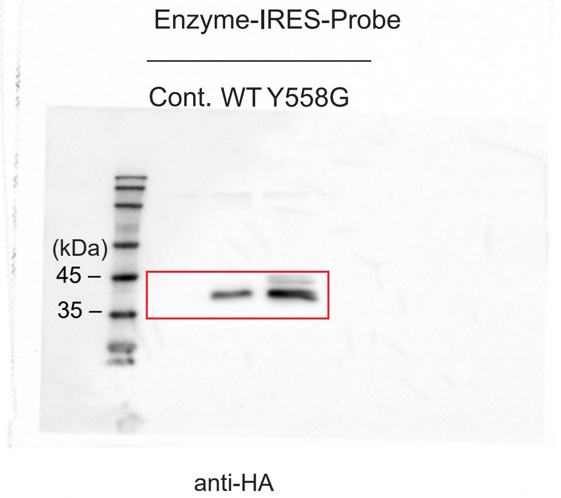

Fig. S3

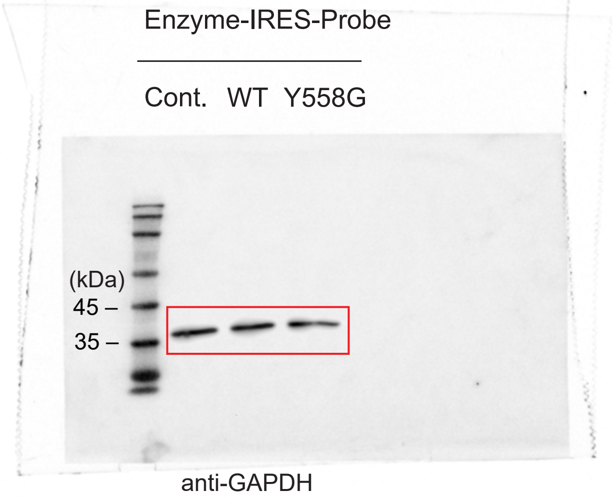

Full western blots and gels in Fig. S1 and S3.
